# Subtle clinical signs of a meningioma in an adult: a case report

**DOI:** 10.1186/2045-709X-22-8

**Published:** 2014-02-04

**Authors:** Andrée-Anne Marchand, Julie O'Shaughnessy

**Affiliations:** 1 Canadian Memorial Chiropractic College (CMCC), Toronto, Ontario and Université du Québec à Trois-Rivieres (UQTR), Trois-Rivieres, Québec, Canada; 2 Département de chiropratique, Université du Québec à Trois-Rivières (UQTR), Trois-Rivières, Québec, Canada

**Keywords:** Meningioma, Brain tumor, Chiropractic, Cranial nerve, Neurological dysfunction

## Abstract

**Background:**

Meningiomas are the most common brain tumor in the adult population. This case report describes the epidemiology, the clinical presentation as well as the current treatment options for this condition.

**Case presentation:**

A 49 year-old man attended a chiropractic clinic with non-specific chronic low back pain. Upon the history taking and the systems review, he reported a loss of both smell and taste for which investigations conducted by two different otolaryngologists did not yield a specific diagnosis. The patient was referred to a neurologist who ordered a computer tomography scan that eventually revealed a compression brain tumor.

Brain tumors can produce a large variety of clinical presentations, such as upper motor neuron lesion symptoms, altered consciousness or vital functions which are easy to identify. However, subtle signs, such as those presented in this case, can be neglected.

**Conclusion:**

Clinicians should be aware of uncommon clinical presentations including cranial nerve or neurological dysfunction and refer their patient to a specialist when detected.

## Abstract (fr)

**Résumé (French language):**

**Introduction**

Les méningiomes sont les tumeurs cérébrales les plus fréquemment rencontrées dans la population adulte. Cette étude de cas décrit l’épidémiologie, la présentation clinique ainsi que les options de traitement pour cette condition.

**Présentation du cas**

Un homme de 49 ans, s’est présenté à une clinique chiropratique ayant comme motif principal une lombalgie chronique non spécifique. L’anamnèse et la revue des systèmes ont révélé une perte de l’odorat et du goût, pour laquelle des examens menés par deux otolaryngologistes différents n’ont mené à aucun diagnostic précis. Le patient fut ensuite référé à un neurologue qui a ordonné une tomographie axiale révélant une tumeur compressive localisée au cerveau.

Les tumeurs cérébrales peuvent être responsables d’une grande variété de manifestations cliniques incluant des symptômes facilement reconnaissables, tels qu’une lésion des motoneurones supérieurs et une altération de la conscience ou des fonctions vitales. Toutefois, des signes plus subtils, tels que ceux présentés dans ce cas, peuvent être négligés.

Les cliniciens devraient être familiers avec les présentations cliniques inhabituelles liées aux méningiomes, y compris les désordres neurologiques. La référence des patients à un spécialiste est souhaitable aussitôt la problématique découverte.

## Background

Meningiomas arise from arachnoidal cells of the leptomeninges and are the most common primary tumors of the central nervous system in adults [1]. Over the past decades, improved and more frequent use of brain imaging has resulted in an increase in the diagnostic incidence and prevalence of meningiomas [1]. According to the United States Central Brain Tumor Registry, meningiomas had the highest incidence and accounted for 35.5% of all primary brain tumors between 2005 and 2009 [2]. Age-adjusted incidence is more than twice as common in females and increases dramatically after age 65 [2]. Ninety–eight percent of meningiomas are reported to be non–malignant and are usually confirmed by either histological or radiographical studies [2]. The following case report highlights the importance of primary contact practitioners awareness to the presence of abnormal symptoms related to meningiomas. It also presents the crucial clinical findings that warrant referral for further investigation.

## Case presentation

### History

A 49 year-old male attended a chiropractic clinic with a history of nonspecific chronic low back pain. Upon further questioning, the patient mentioned a progressive decrease in sense of smell and taste which was reported as complete since the last two and a half years. Following medical consultation for this matter since a year ago, two otolaryngologists had been consulted and both conducted nasal and oesophageal endoscopies that were considered normal. Lifestyle review revealed an average consumption of four alcoholic beverages daily and sometimes up to forty-eight to seventy-two beverages weekly. The result of the CAGE questionnaire [3] which yielded two positive answers (need to cut down on drinking (C) and need a drink first thing in the morning (E: eyes opener)) out of four (annoyed by people criticizing your drinking (A) and ever felt guilty about drinking (G)) was in accordance with the patient acknowledgement of his alcohol problem. Past medical history revealed bilateral knee meniscectomy, right carpal tunnel surgical decompression, L4-L5 discectomy and excision of a benign cervical lipoma. The patient reported his health status as otherwise healthy and denied the presence of any constitutional symptoms.

### Physical examination

Upper and lower limb neurological examination was bilaterally present, symmetric and rated as unremarkable for deep tendon reflexes, sensation and motor strength. Pathological reflexes were absent bilaterally. A cranial nerve exam revealed anosmia. In accordance with diagnostic imaging practice guidelines [4] cervical spine radiographs were ordered in light of the presence of central nervous system sign and symptoms. The radiological examination revealed postural anomalies, cervical degenerative disc disease and congenital non-union of the posterior arch of the atlas. Given the patient’s age, his non-smoking status, absence of trauma, unremitting nature of the symptoms and lack of previous invasive investigation the chiropractor suspected a neurological compression from internal cause. The decision was made to refer the patient to a neurologist for further investigation of the decreased sense of smell and taste.

### Intervention and outcome

**Figure 1:**
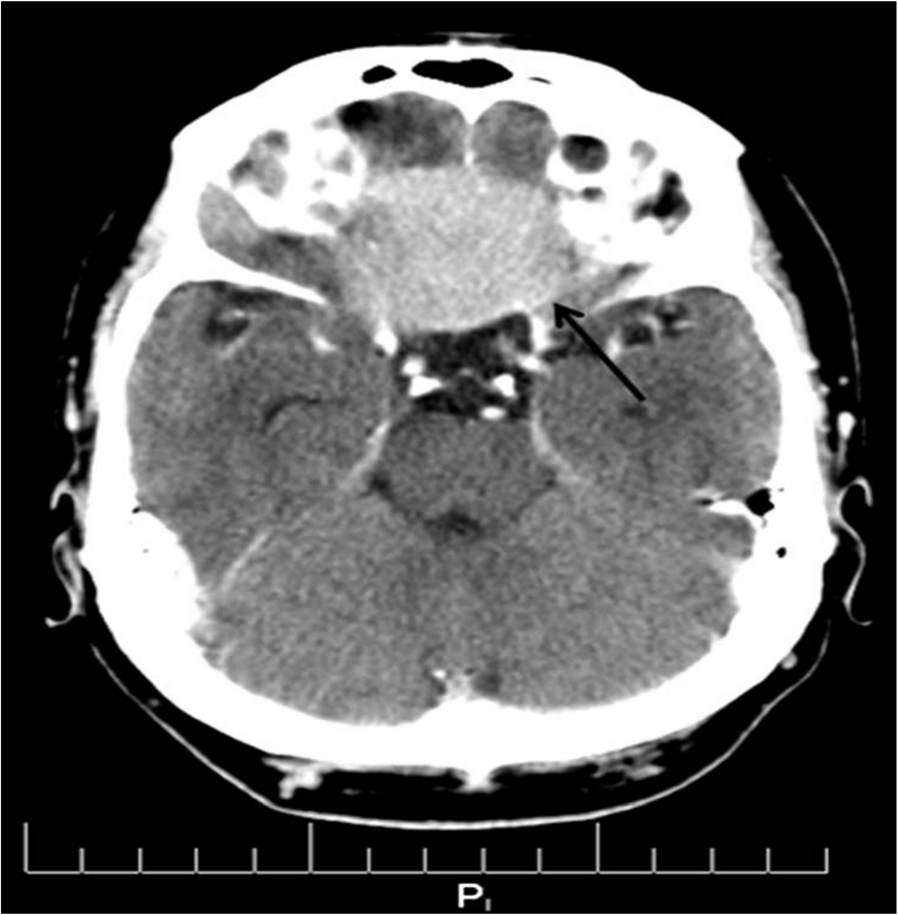
**Axial view, CT of the head, soft tissue window reveals a 6x6 cm well marginated meningioma extending into the planum sphenoidale.**

**Figure 2:**
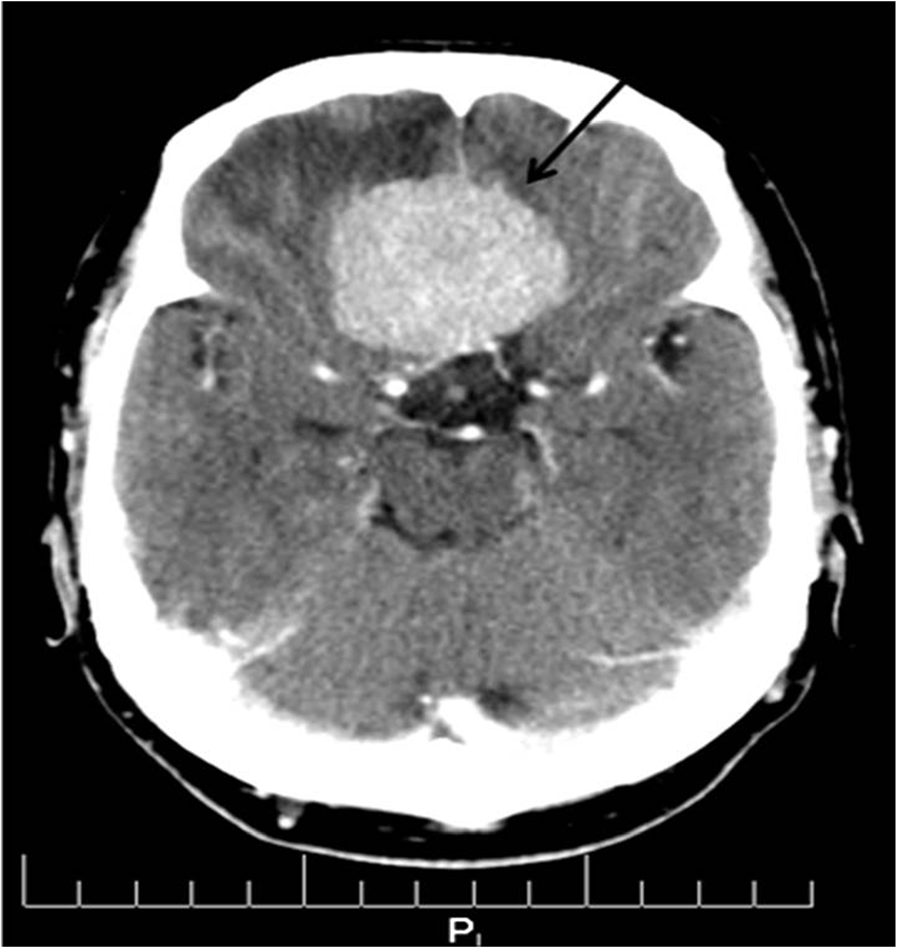
**Axial view, CT of the head, soft tissue window reveals compression of the frontal lobe by the meningioma.**

A computer tomography (CT) scan ordered by the neurologist upon initial assessment revealed a 6 cm planum sphenoidale mass located in the frontal lobe, suggesting a benign meningioma (Figures 1 and 2). The tumor was well circumscribed and no osseous abnormalities were detected. The patient underwent a frontolateral craniotomy with complete resection of the tumor mass. Post-surgical complication of a cerebrospinal fluid (CSF) leakage through the nasal cavity occurred 3 days later. A spinal tap was initially used to drain the CSF, but subsequent air accumulation within the intracranial cavity as visualized on CT led to a second surgical intervention. The dural breach was repaired through reconstruction of the anterior base of the ethmoid. No other surgical complication was reported and the pathological analysis confirmed a diagnosis of meningioma.

At one year follow-up, the patient did not report any neurological symptoms, except anosmia. No further surgical complications were reported. He had no limitations to his daily living activities and he returned back to work full time. Of interest, the patient’s problem with alcohol resolved without treatment after the surgery.

## Discussion

Meningiomas of the midline anterior skull base such as the olfactory groove meningioma presented in this case report are a rare clinical entity and represent about 10% of all intracranial meningiomas [5]. Olfactory groove meningioma (OGM) originates from the anterior cranial base, commonly at the cribriform plate of the ethmoid bone, planum sphenoidale or the frontospenoidal suture [6]. Arising from the weakest part of the skull base makes it prone to infiltration of the underlying bone. It also sometimes extend into the paranasal sinuses and nasal cavity, displacing the olfactory tracts laterally and the optic chiasm posteriorly [7]. The tumors are generally encapsulated and attached to the dura. OGMs receive their vascular supply primarily from the anterior and posterior ethmoidal arteries. Vascular contribution from the dura, anterior branches of the middle meningeal artery and the meningeal branches of the ophthalmic artery are also often seen [8].

Although there are several meningioma subtypes, (meningotheiomatous, fibrous, psammomatous) their identification has little prognostic value. Only clear cell meningiomas are thought to have clinical significance due to their aggressive behaviour [8]. Histologic features are used to classify meningiomas into one of the three World Health Organization (WHO) grades and are useful in predicting recurrence and survival rate. About 90% of tumors are grade I (benign) with a risk of recurrence after post-surgical resection of 7% to 20%. Incidence of grade II meningiomas (atypical) is 5% to 7%, with a recurrence rate of 40% whereas less than 3% are classified as grade III (malignant or anaplastic). Between 50% to 80% of malignant cases will recur [9].

Several risk factors for the development of meningiomas have been hypothesized, and until now, the literature does not reveal any clear association. Environmental risk factors such as exposure to ionizing radiation are currently acknowledged as the leading cause underlying meningioma formation [8]. As such, low dose exposure as it is the case in dental x-rays, is a common source of radiation in the general population. Increased risks of developing meningioma have been identified with having had a bitewing examination at any age (Odd ratio: 2.0) and panoramic films under the age of 10 years old (Odd ratio: 4.9) [10]. A population-based prospective cohort study which included 27,791 post-menopausal women has determined that low levels of physical activity, history of oophorectomy (unilateral or bilateral), high height and body mass index (BMI) in the years preceding diagnosis and history of uterine fibroids were associated with an increased risks of meningioma [11]. In a large case–control study examining the relationship between family, personal medical history and meningioma, the results suggested a positive association between meningioma and first-degree family members diagnosed at a young age, female related hormone and ionization radiation [12].

### Clinical presentation

Although they usually remain clinically quiescent during the early phase of growth, meningiomas localized to the frontal lobe result in a variety of alterations in cognitive and functioning behaviours [5]. The most common symptoms reported are headaches, visual disturbances and anosmia, often leading to incorrect diagnosis such as sinusitis, migraine and neuralgia [13]. In a retrospective study of 56 cases of olfactory groove meningioma, preoperative signs and symptoms screening revealed mental changes, headache, visual disturbance, dizziness, seizure, papilledema and hemiparesis [7]. Of interest in our present case, the patient reported spontaneous resolution of his alcoholism following the surgical intervention. To the best of our knowledge no previous report of a possible association between meningioma and alcoholism exits in the literature. Although other factors could explain the sudden patient’s sobriety, it seems unlikely that heavy drinking habits could have stopped without any withdrawal effects. Given that tumours located in the frontal region are responsible for mental alterations such as attention deficits, disinterest and emotional detachment we hypothesize that alcoholism may have been the initial symptom experienced by the patient as a result of the meningioma growth.

### Physical examination

Within the primary care setting, the suspicion of olfactory groove meningioma may be based solely on information gathered in the history. Although anosmia is thought to be an early symptom, surprisingly few patients complain of olfactory dysfunction, making its detection extremely difficult during routine clinical examination. Proposed explanations for the lack of suspicion include natural gradual decline in olfactory function and lateralised anosmia in which case olfactory function is maintained by the contralateral side [14]. When particular attention is brought to the olfactory function, the “sniffin’ sticks” test can be performed to identify any odour threshold, discrimination, and identification deficits [14]. It has been suggested that in order to promptly diagnose at an early stage, CT scan “should be used in all patients presenting with loss of sense of smell that cannot be explained by head injury, other disease or previous surgical procedures of the olfactory region” [13]. Physical examination should include nasal endoscopy, as up to 15% of patients with OGMs are reported to have an intranasal component [15].

### Imaging

Diagnosis and decision on the best surgical approach are usually made with the use of magnetic resonance imaging, due to its ability to show the tumor’s dural origin, along with perifocal edema and location or encasement of major vessels [16]. In addition, meningiomas display a strong homogeneous enhancement when gadolinium contrast is used. Images displayed by CT show meningiomas as well-defined extra-axial masses associated with brain displacement and allow for identification of characteristics such as intratumoral calcification or hyperostosis [8,16].

### Treatment

Treatment options for OGMs follow those in other skull base tumors. It is generally accepted that a small tumor, stable in size over time and asymptomatic, should be monitored through serial imaging. However, for meningiomas occurring in patients younger than 65 years of age, surgery is usually recommended. Curative treatment of meningiomas can usually be achieved by surgery alone when the tumor, its dural attachment and infiltrated bone can be completely resected. Even large size tumors can be totally removed due to the arachnoid membrane creating a separation from nearly all surrounding critical neurovascular structures [8]. Despite numerous surgical approaches that have been described in the literature and the refinement of technologies, removal of meningiomas of the anterior cranial base remains a challenge due to complex anatomical relations and surrounding of important neurovascular structures. One common theme discussed in the literature is that regardless of the technique used and tumor size, preservation of olfactory or visual function ipsilateral to the location of the tumor has a very poor outcome.

Radiation therapy comes into play in the management of meningioma when gross total resection cannot be safely achieved, such as in optic nerve sheath meningiomas, when tumors recur after surgery and in radiographically diagnosed tumors when biopsy is not possible. Radiotherapy is also provided as adjuvant therapy following resection of atypical and malignant meningiomas [8,9]. The role of chemotherapy is limited to treatment of tumors that recur after surgery and only when radiotherapy options are exhausted due to its reported minimal impact against this type of neoplasm [9].

### Prognosis

When OGM resection results were initially reported, mortality ranged from 17.3% to 22.7% [8]. Recent literature revealed decreased mortality associated to meningioma with a 3-year post diagnosis survival estimate of 93.4% in patients treated with resection compared to 88.3% in patients not surgically treated. Younger patient age, female gender, unilateral tumors and surgical resection appear to be potential predictors of improved prognosis in patients with meningiomas [17]. The recurrence rate at 10 years has been reported to vary from 9% to 20% for totally resected meningiomas, and from 18.4% to 50% for meningiomas following subtotal resection. The mean time to recurrence of meningiomas has been determined to be 2.5 years to 5 years [18]. Although limited evidence is available on the prognosis of untreated meningiomas, low growth rate tumors have been reported not to be an obstacle to patients function and well being even in presence of slight neurological symptoms [19].

## Conclusion

Although meningioma is the most common primary tumor in the nervous system in adults, it is a rare condition. In the presence of subtle clinical signs such as those presented in this case report, clinicians should keep a high index of suspicion for brain tumor. In the presence of uncommon neurological signs and symptoms, cranial nerve dysfunction or failed conservative therapy, clinicians should not hesitate to refer the patient for further evaluation as part of a complete management program.

### Informed consent

Written informed consent was obtained from the patient for publication of this Case report and any accompanying images. A copy of the written consent is available for review by the Editor-in-Chief of this journal.

## Competing interest

The authors declare that they have no competing interests.

## Authors’ contributions

AAM and JO contributed to the literature review and writing of the manuscript. All authors read and approved the final manuscript.

## References

[B1] Saraf S McCarthy BJ Villano JL Update on meningiomas Oncologist 2011 16 1604 1613 10.1634/theoncologist.2011-0193 22028341 PMC3233296

[B2] Dolecek TA Propp JM Stroup NE Kruchko C CBTRUS statistical report: primary brain and central nervous system tumors diagnosed in the United States in 2005–2009 Neuro Oncol 2012 14 Suppl 5 v1 v49 10.1093/neuonc/nos218 23095881 PMC3480240

[B3] Ewing JA Detecting alcoholism. The CAGE questionnaire JAMA 1984 252 1905 1907 10.1001/jama.1984.03350140051025 6471323

[B4] Bussieres AE Taylor JA Peterson C Diagnostic imaging practice guidelines for musculoskeletal complaints in adults-an evidence-based approach-part 3: spinal disorders J Manipulative Physiol Ther 2008 31 33 88 10.1016/j.jmpt.2007.11.003 18308153

[B5] Gazzeri R Galarza M Gazzeri G Giant olfactory groove meningioma: ophthalmological and cognitive outcome after bifrontal microsurgical approach Acta Neurochir (Wien) 2008 150 1117 1125 10.1007/s00701-008-0142-z 18936875

[B6] Pepper JP Hecht SL Gebarski SS Lin EM Sullivan SE Marentette LJ Olfactory groove meningioma: discussion of clinical presentation and surgical outcomes following excision via the subcranial approach Laryngoscope 2011 121 2282 2289 10.1002/lary.22174 21994142

[B7] Bassiouni H Asgari S Stolke D Olfactory groove meningiomas: functional outcome in a series treated microsurgically Acta Neurochir (Wien) 2007 149 109 121 10.1007/s00701-006-1075-z 17180303

[B8] Adappa ND Lee JY Chiu AG Palmer JN Olfactory groove meningioma Otolaryngol Clin North Am 2011 44 965 980 ix 10.1016/j.otc.2011.06.001 21819883

[B9] Norden AD Drappatz J Wen PY Advances in meningioma therapy Curr Neurol Neurosci Rep 2009 9 231 240 10.1007/s11910-009-0034-5 19348712

[B10] Claus EB Calvocoressi L Bondy ML Schildkraut JM Wiemels JL Wrensch M Dental x-rays and risk of meningioma Cancer 2012 118 4530 4537 10.1002/cncr.26625 22492363 PMC3396782

[B11] Johnson DR Olson JE Vierkant RA Hammack JE Wang AH Folsom AR Virnig BA Cerhan JR Risk factors for meningioma in postmenopausal women: results from the Iowa Women’s Health Study Neuro Oncol 2011 13 1011 1019 10.1093/neuonc/nor081 21750006 PMC3158016

[B12] Claus EB Calvocoressi L Bondy ML Schildkraut JM Wiemels JL Wrensch M Family and personal medical history and risk of meningioma J Neurosurg 2011 115 1072 1077 10.3171/2011.6.JNS11129 21780859 PMC3241000

[B13] Tsikoudas A Martin-Hirsch DP Olfactory groove meningiomas Clin Otolaryngol Allied Sci 1999 24 507 509 10.1046/j.1365-2273.1999.00303.x 10606998

[B14] Welge-Luessen A Temmel A Quint C Moll B Wolf S Hummel T Olfactory function in patients with olfactory groove meningioma J Neurol Neurosurg Psychiatry 2001 70 218 221 10.1136/jnnp.70.2.218 11160471 PMC1737227

[B15] Derome PJ Guiot G Bone problems in meningiomas invading the base of the skull Clin Neurosurg 1978 25 435 451 710009 10.1093/neurosurgery/25.cn_suppl_1.435

[B16] Rachinger W Grau S Tonn JC Different microsurgical approaches to meningiomas of the anterior cranial base Acta Neurochir (Wien) 2010 152 931 939 10.1007/s00701-010-0646-1 20383724

[B17] Cahill KS Claus EB Treatment and survival of patients with nonmalignant intracranial meningioma: results from the surveillance, epidemiology, and end results program of the National Cancer Institute. Clinical article J Neurosurg 2011 115 259 267 10.3171/2011.3.JNS101748 21529132 PMC3256743

[B18] Snyder WE Shah MV Weisberger EC Campbell RL Presentation and patterns of late recurrence of olfactory groove meningiomas Skull Base Surg 2000 10 131 139 10.1055/s-2000-9316 17171137 PMC1656819

[B19] Bindal R Goodman JM Kawasaki A Purvin V Kuzma B The natural history of untreated skull base meningiomas Surg Neurol 2003 59 87 92 discussion 92 10.1016/S0090-3019(02)00995-3 12648902

